# Health and Wellbeing of Occupants in Highly Energy Efficient Buildings: A Field Study

**DOI:** 10.3390/ijerph14030314

**Published:** 2017-03-19

**Authors:** Peter Wallner, Peter Tappler, Ute Munoz, Bernhard Damberger, Anna Wanka, Michael Kundi, Hans-Peter Hutter

**Affiliations:** 1 Institute of Environmental Health, Center for Public Health, Medical University Vienna, Kinderspitalgasse 15, 1090 Vienna, Austria; peter.wallner4@gmail.com (P.W.); michael.kundi@meduniwien.ac.at (M.K.); 2 Austrian Institute for Healthy and Ecological Building, Alserbachstraße 5, 1090 Vienna, Austria; p.tappler@innenraumanalytik.at (P.T.); u.munoz@innenraumanalytik.at (U.M.); b.damberger@innenraumanalytik.at (B.D.); 3 Institute of Sociology, University of Vienna, Rooseveltplatz 2, 1090 Vienna, Austria; anna.wanka@univie.ac.at

**Keywords:** energy efficient buildings, housing, indoor air quality, mechanical ventilation, natural ventilation, self-reported health, perception

## Abstract

Passive houses and other highly energy-efficient buildings need mechanical ventilation. However, ventilation systems in such houses are regarded with a certain degree of skepticism by parts of the public due to alleged negative health effects. Within a quasi-experimental field study, we investigated if occupants of two types of buildings (mechanical vs. natural ventilation) experience different health, wellbeing and housing satisfaction outcomes and if associations with indoor air quality exist. We investigated 123 modern homes (test group: with mechanical ventilation; control group: naturally ventilated) built in the years 2010 to 2012 in the same geographic area and price range. Interviews of occupants based on standardized questionnaires and measurements of indoor air quality parameters were conducted twice (three months after moving in and one year later). In total, 575 interviews were performed (respondents’ mean age 37.9 ± 9 years in the test group, 37.7 ± 9 years in the control group). Occupants of the test group rated their overall health status and that of their children not significantly higher than occupants of the control group at both time points. Adult occupants of the test group reported dry eyes statistically significantly more frequently compared to the control group (19.4% vs. 12.5%). Inhabitants of energy-efficient, mechanically ventilated homes rated the quality of indoor air and climate significantly higher. Self-reported health improved more frequently in the mechanically ventilated new homes (*p* = 0.005). Almost no other significant differences between housing types and measuring time points were observed concerning health and wellbeing or housing satisfaction. Associations between vegetative symptoms (dizziness, nausea, headaches) and formaldehyde concentrations as well as between CO_2_ levels and perceived stale air were observed. However, both associations were independent of the type of ventilation. In summary, occupants of the mechanically ventilated homes rated their health status slightly higher and their health improved significantly more frequently than in occupants of the control group. As humidity in homes with mechanical ventilation was lower, it seems plausible that the inhabitants reported dry eyes more frequently.

## 1. Introduction

Very energy-efficient homes, such as passive houses, are those which meet rigorous energy efficiency standards. Because of the air tightness, such buildings need built-in mechanical ventilation [1]. Heat recovery systems are necessary in order to minimize energy loss [1,2]. There are, however, concerns that such ventilation systems may impact health through exposure to excess noise, draughts, and indoor air pollution as a consequence of insufficient cleaning of the air duct system and low levels of indoor air humidity due to an increased volume of outdoor air in winter [2,3,4]. Energy-efficient homes without mechanical ventilation have also been found to be associated with an increased risk of asthma in the United Kingdom [5]. However, a meta-analysis by Maidment and colleagues concluded that energy efficiency interventions led to a small but statistically significant improvement in the health of residents [6].

In a study with more than 3000 measurements (chemical pollutants, biological contaminants, indoor climate parameters) we found that the indoor air quality in highly energy-efficient, mechanically ventilated homes was higher than that of conventional homes [7]. Pollutant concentrations in French low-energy school buildings with ventilation systems were lower than in conventional school buildings [8]. A few studies reported that ventilation systems in homes lead to a reduction in reported health symptoms and improvements in overall health [9,10,11], likely attributable to an increased air exchange and thus an improvement in indoor air quality.

Leech et al. [9] examined self-reported changes in health status by telephone-administered questionnaires in occupants of new homes in Canada. Occupants of the test group (energy-efficient homes with heat recovery ventilators) provided a health benefit over one year of occupancy.

A study in Cornwall, UK (The Breath of Fresh Air Project), investigated the health of asthmatic children in 17 homes [10]. Indoor measurements and health assessments (by questionnaires) were conducted before and after the installation of mechanical ventilation and heat recovery (MHRV) systems. Installations of MHRV systems reduced mite allergen concentrations and children’s asthma symptoms.

After “green” renovation (installation of mechanical ventilation, tightening of the building envelope, etc.) of low-income housing in Minnesota, participants’ health and building performance were assessed [11]. Health was assessed via questionnaire. Interviews were administered after residents moved into renovated apartments and approximately 12 to 18 months later. The renovation produced improvements in health, and energy use was reduced by 45% over the one-year period.

In this paper we compared the self-rated health and wellbeing of inhabitants of very energy-efficient homes to the health of inhabitants of conventional new houses without mechanical ventilation. In addition, we also evaluated the participants’ perception of the indoor air quality (e.g., stale air as a consequence of increased CO_2_ or smells) and indoor climate (temperature, humidity, air movement) and their housing satisfaction.

## 2. Materials and Methods

Inhabitants of new houses built according to very low energy or passive house standards (Austrian Standard B 8110-1) [12] formed the test group. The houses had no air conditioning. Inhabitants of houses which corresponded to the normal building standards without mechanical ventilation systems formed the control group. It was assumed that in the buildings of the test group the air supply was provided both mechanically and via ventilation through windows (and doors). The study was approved by the ethics committee of the Medical University of Vienna (377/2010).

Recruitment of participants is described in [7]. The buildings were located in all provinces of Austria and were built between 2010 and 2012. In both groups, detached houses constituted approximately 70% of the sample. The remaining 30% were apartments in multistory buildings.

Interviews were conducted at two different time points (first interview and follow-up interview). Also measurements of indoor parameters (climate, chemical pollutants and biological contaminants) were conducted twice according to standardized analytical methods (e.g., formaldehyde according to ISO 16000-2 and 3 [13,14]). Methods and results of the measurements were reported in [7].

The first interview (measurement point T1, *n* = 293, between October 2010 and May 2012) occurred at approximately three months (±3 weeks) after moving into a new house/apartment, with a follow-up interview (measurement point T2, *n* = 282, between October 2011 and May 2013) one year later. The drop-out rate was 4%.

Interviews were conducted with a structured questionnaire. The questionnaire consisted of the standardized questionnaire SF-36 (36-Item Short Form Survey, [15]) and a section of the wellbeing questionnaire used in AUPHEP (Austrian Project on Health Effects of Particulates, [16]). It consisted of the following parts: Socio-demographic characteristics of the participants, respiratory symptoms and allergies, unspecific symptoms; perception of indoor air quality and climate; satisfaction with the housing situation.

### Statistical Analysis

Collected data were analysed using SPSS 20.0 for Windows (SPSS Inc., Chicago, IL, USA). Comparisons of categorical data across groups were done by chi-square tests, comparison of time points within groups were done by Bowker’s symmetry tests. Symptom ratings were combined into scores (psychasthenic symptoms, vegetative symptoms). Also air quality ratings were combined into scores with positive attributes (fresh, clean, pleasant, fragrant) into one and negative (stale, stuffy, stagnant, bad smelling, smoky) into another score. These scores as well as climate ratings were McCall transformed (standardized scores: mean 0, standard deviation 1) and subjected to analyses of covariance with group as between subjects and time points as within subjects factor and gender and age as covariates. For the analyses of relationships between ratings and measurements a log transformation of air quality and climate data was performed and linear regression analysis including age and gender as potential confounders was done. For all analyses *p*-values below 0.05 were considered significant.

## 3. Results

In total, 575 interviews (test group: 299, control group: 276) were conducted between October 2010 and May 2013 (first time point: *n* = 293, second time point: *n* = 282). Of these, 409 interviews were conducted with adults. Parents also filled in questionnaires for the 166 (86 control group, 80 test group) children (<16 years of age) included.

The average age of adults in both test and control groups at T1 was virtually the same (37.9 ± 9 years in the test group, 37.7 ± 9 years in the control group); children were, on average, 5.7 years of age in the test group and 7.5 years in the control group. The average household size included 2.8 ± 1.1 participants in both groups; most households consisted of couples and included, on average, 0.8 children <16 years of age (for both the test and control groups).

Smokers accounted for 18.4% in the test group and 25.4% in the control group. Of these, only 0.5% (test group) and 2.6% (control group) smoked in their apartment or house.

Due to the relatively high costs of such homes, all participants belonged to the upper-middle class (more than 12 years of education, household income above median).

### 3.1. Subjective State of Health

There were slight differences by housing type for both adults and children in health ratings. Participants in the test group rated their own health and that of their children higher compared with the control group: 24.9% (average of the ratings at T1 and T2) considered themselves and 50.6% considered their children to be in excellent health, compared with 19.8% and 38.4%, respectively, in the control group (Table 1 and Table 2).

Table 1 also shows the health ratings before moving in (recall at T1). In adults, there was a trend towards improvement in the state of health after moving in, but this was not statistically significant. After participants had been living in their new home for more than one year (T2), there was barely any change in the state of health in comparison to T1.

The children’s state of health was rated only twice, three months after moving in at T1 and one year later at T2 (Table 2). There was a marked difference over time in the control group (*p* < 0.05): in this group, parents perceived their children’s health to be considerably better one year after moving in, compared with a downwards shift from “excellent” to “very good” in the test group. In both groups, “good and less good” ratings changed to a more positive perception over time.

In addition, the participants rated their change in health status over the last year at T2. At this time (T2), participants had already been living at their new address for approximately one year. Of adults who moved into new housing with a mechanical ventilation system, 19.1% experienced improvements in their health, while 80% saw no change and 0.9% noted some deterioration. In contrast, 13.2% of adults who had moved into buildings without mechanical ventilation perceived that their health had deteriorated, 69.2% felt no change and 17.6% noted some improvement. This difference was statistically significant (*p* = 0.005).

When adult participants were asked to predict their future state of health, 4.3% of the test group and 7.9% of the control group believed that their health would likely deteriorate.

### 3.2. Allergies

In total, 29.9% of all participants reported having allergies; 33.6% of adults and 12.7% of children in the test group and 37.5% of adults and 17.8% of children in the control group were affected. Adults in the test group suffered from an average of two allergies, compared with an average of 2.1 in the control group. The average number of allergies in children was one in the test group and 1.5 in the control group. The most common types were pollen allergies (32.6%), followed by pet hair allergies (23.3%), dust mite allergies (22.2%) and food allergies (16.1%).

There was no significant difference in the number or frequency of allergies in residences with mechanical ventilation systems; however, allergies against pollen, pet hair and insects were less frequently observed in occupants with mechanical ventilation (*p* < 0.05). Prevalence, type or number of allergies did not change over time in either group.

### 3.3. Respiratory Complaints and Eye Problems

In total, 35.5% of the participants experienced, within the four weeks before measurement, dryness of the airways, 22.6% felt a burning sensation in their nose or throat and 13.9% had dry, red or itchy eyes without having a coinciding cold or having visited a swimming pool. Further, 12.5% of participants had been coughing for more than two weeks within the preceding four weeks of the interview.

There were no significant differences by housing type in prevalence and number of colds, dryness of the airways, burning sensations in the nose or throat, or coughs; however, adults in the test group had a significantly higher prevalence of dry eyes (19.4%) compared to the control group (12.5%), independent of contact lens usage (*p* = 0.04). There were no significant changes in the evaluated health complaints in either group over time.

### 3.4. Other Health Impairments

Participants were asked how often they experienced the following symptoms in the last four weeks: tiredness, exhaustion, headaches, nausea, dizziness, impaired concentration, anxiety, nervousness, mood changes, and limited performance. The results are shown in Table 3.

Neither adults nor children showed any differences by type of residence. Children were generally less affected by the listed health impairments compared with adults.

In the test group, there was an increase in tiredness, exhaustion and nervousness after one year; in the control group, increased difficulty to concentrate and nervousness were observed after one year. These differences did not reach statistical significance. The average numbers of health impairments were similar in both groups (Table 3).

### 3.5. (Parental) Perception of Air Quality, Climate, Smell and Noise at Home

Negative perceptions of the quality of air (stale, stuffy, stagnant, bad smelling, smoky) were found more frequently in homes without mechanical ventilation. Differences in negative perceptions of indoor air quality between the groups were overall highly significant (*p* < 0.01), with the exception of “bad smelling” and “smoky”. The results are shown in Table 4.

The difference in the positive perception of air quality between groups was highly significant for the attributes “pleasant” and “fresh” (*p* < 0.01), and significant for the attribute “clean” (*p* < 0.05). In all these cases, positive perception was more frequent in homes with ventilation systems (Table 5). Air quality ratings did not significantly change in the period between measuring points.

The perception of indoor climate, smell and noise is presented in Table 6. No significant differences between measurement points were found, with the exception of satisfaction with humidity in the test group, which 63.9% of participants rated as “just right” at T1, compared with only 53.6% at T2. Therefore, we only report here the average perception (T1 and T2) of indoor climate over one year. Participants who lived in housing with mechanical ventilation (test group) rated temperature and air movement in their homes as significantly more pleasant (*p* < 0.01) compared with the control group: 77.0% of participants in the test group and 65.2% in the control group rated their room temperature as “just right”; 7.9% and 12.1%, respectively, rated it as “(too) cold”; and 15.2% and 22.7%, respectively, as “(too) warm”. Air movement was considered to be “just right” by 80.6% of participants in the test group and 66.7% in the control group. More participants in the control group compared with the test group complained about draught (29.1% vs. 14.3%, respectively).

The control group rated the humidity in their home significantly better compared with the test group: 58.8% of participants in the test group considered the humidity to be “just right”, compared with 67.2% in the control group (*p* < 0.01); 40.6% of participants in the test group thought that the air at their home was (too) dry, compared with 26.4% in the control group.

There were no significant differences regarding annoyance due to smells or noise between groups. Between 48.2% and 56.1% rated smell and noise as “not annoying at all”.

### 3.6. Satisfaction with the Housing Situation

Most participants felt that their current housing situation had improved significantly compared with their previous housing situation. Accordingly, 80.6% of participant who lived in housing with mechanical ventilation and 72.0% of participants of the control group felt much more satisfied with their housing conditions; 12.0% and 16.9%, respectively, felt rather more satisfied and 7.4% and 11.0%, respectively, felt neither more nor less satisfied, or dissatisfied.

Satisfaction with the housing situation around the time of T1, three months after moving in was completed, was particularly high in the test group: 86.9% were very content with their housing situation, 10.3% were content and only 2.8% were neither content nor not content, or dissatisfied (Table 7). In the control group, 76.5% were very content at the time of T1, 21.4% were content and 2.0% were neither content nor not content, or dissatisfied. Differences in the satisfaction at the time of T1 were not statistically significant.

At the point of T2 (one year after T1), there was only a slight reduction in the level of satisfaction (Table 7).

With regard to the neighborhood conditions, the following observations were made: At the time of T1, 70.1% of participants in the test group declared that they were very satisfied with their neighborhood conditions, 25.2% were satisfied and 3.7% were dissatisfied or neither satisfied nor dissatisfied, compared with 72.4%, 24.5% and 3%, respectively, in the control group. At the time of T2, 68.2% in the test group and 64.8% in the control group were found to be very satisfied with their neighborhood conditions; 26.4% and 31.9%, respectively, were satisfied and 4.4% and 3.3%, respectively, were dissatisfied or neither satisfied nor dissatisfied. None of these findings indicated statistical significance between groups.

Participants in the test group perceived themselves to be significantly more satisfied than their peer group, compared with the control group. Further, 64.1% of participants in the test group and 54.0% participants in the control group estimated that, compared with family and friends, they were much more content with their living situation; 26.3% and 27.5%, respectively, were rather more content, 7.4% and 17.5%, respectively, were equally content and 2.2% and 1.0%, respectively, considered themselves to be more dissatisfied (*p* < 0.01).

### 3.7. Correlations between Subjective Experiences and Measurements of Air Quality and Climate

**Figure 1:**
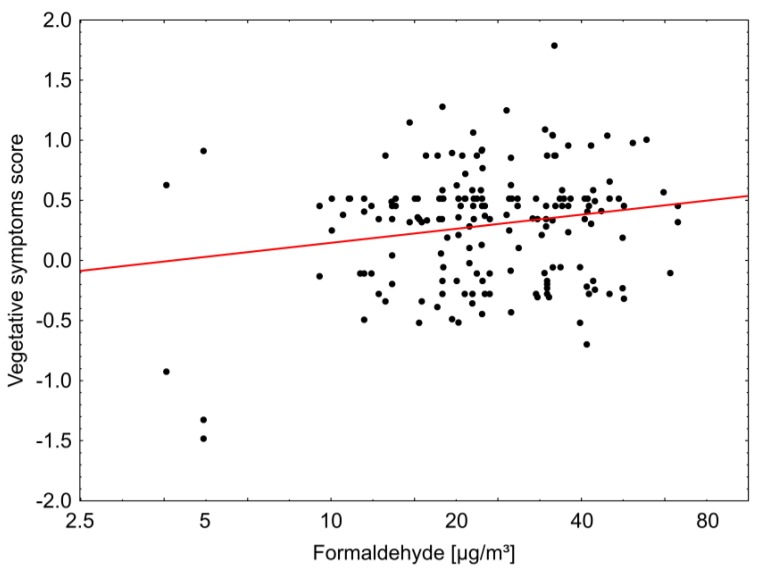
Correlation between indoor concentration of formaldehyde and frequency of vegetative symptoms (dizziness, nausea, headaches; standardized score) at T2 (about 1.3 years after moving in) (R^2^ = 2.3%).

There was a weak but statistically significant correlation between the frequency of vegetative symptoms (dizziness, nausea, headaches) and the concentration of aldehydes, in particular formaldehyde, at T2 (Figure 1). This correlation was independent of the type of ventilation, although it has to be noted that indoor formaldehyde concentrations in the test group were significantly lower [7].

**Figure 2:**
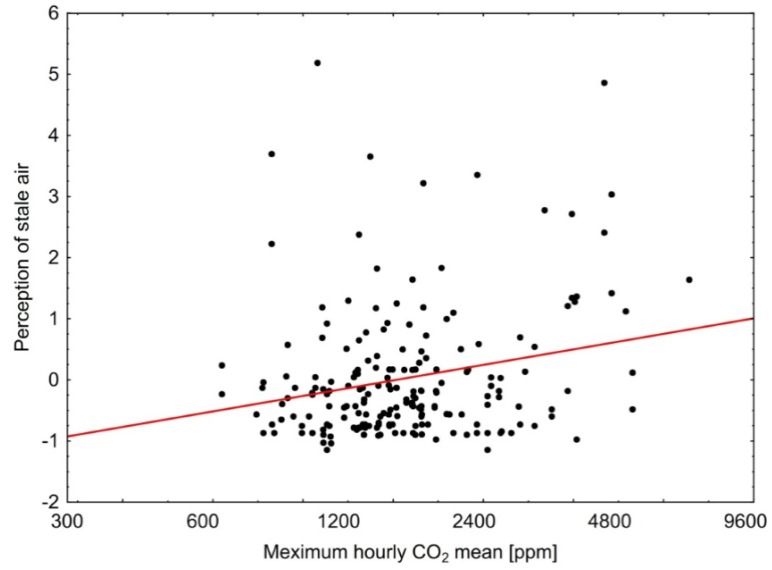
Correlation between maximum hourly mean of indoor CO_2_ concentration (in bedrooms) and perception of stale indoor air (standardized score) at T2 (about 1.3 years after moving in) (R^2^ = 3%).

There was also a significant correlation between the indoor CO_2_ concentration (especially the highest hourly CO_2_ mean value = maximum hourly mean) and the perception of stale indoor air (Figure 2). This correlation was also independent of the study group. No other significant correlations could be found.

## 4. Discussion

To our knowledge this was, besides, inter alia, the investigations of Leech et al. [9] and Takaro et al. [17], one of the first studies investigating the perceived health of inhabitants of highly energy-efficient homes. Our inspiration to conduct the current study was the Canadian study by Leech et al. [9].

Inhabitants of buildings with mechanical ventilation systems in Austria rated their state of health and that of their children slightly higher than participants who lived in dwellings with natural ventilation only.

Furthermore, after about 15 months in their new homes, respondents perceived significantly more frequent improvements over the last year if they had lived in housing with mechanical ventilation. This might, in part, be explained by the better air quality [7] in these homes. Leech et al. [9] also found that new occupants of energy-efficient homes (with ventilation systems) reported an improvement over one year in health in comparison with control home occupants.

No significant differences by housing type or time points were observed regarding the frequency and number of almost all minor ailments or health complaints. Allergies against pollen, pet hair and insects were less frequent in the test group. However, as percentages did not change over time, it seems rather unlikely that the difference in frequency was due to the housing type.

Adults in the test group suffered significantly more frequently from dry eyes compared with adults in the control group. This might be due to the lower humidity in the homes with mechanical ventilation [1]. Accordingly, 40.6% of participants in the test group thought that the air in their home was (too) dry compared with 26.4% in the control group. Measurements also showed that humidity was lower in the test group [7].

We found a weak but statistically significant correlation between the frequency of vegetative symptoms (dizziness, nausea, headaches) and the concentrations of formaldehyde. Such symptoms have been described in the literature also at relatively low levels of formaldehyde, even at or below 0.10 mg/m^3^ [18,19,20]. They may also be associated with other indoor pollutants including CO_2_ (an indicator of adequate ventilation) and smells. However, no such correlations could be found.

Satisfaction with housing and living area in both groups was relatively high: 84.3% of the test group and 76.2% of the control group were very content with their housing; 69.1% and 68.8%, respectively, were very content with the living area. These differences in housing satisfaction between the test and control groups were not significant. However, participants in the test group perceived themselves to be significantly more satisfied with their homes than their peer group, compared with the control group. This may in part be explained by the fact that very energy-efficient homes with energy recovery ventilation systems are still “special” houses in Austria.

According to the results of the measurements in the studied homes [7], there were highly significant differences regarding the subjectively perceived quality of air between both groups, with a perceived higher quality of air in the test group. Temperature and air movement were rated significantly more pleasant in the test group. There were no differences between groups regarding smell and noise exposure.

## 5. Conclusions

In conclusion, inhabitants of new energy efficient buildings with mechanical ventilation generally rated their health and the quality of the indoor air and climate better compared with those who lived in dwellings with window (and doors) ventilation only. However, adults in homes with mechanical ventilation—where humidity was lower [7]—suffered more frequently from dry eyes and found the indoor air (too) dry.

## Figures and Tables

**Table 1:** Subjective health ratings (percentages) before moving in, at T1 (three months after moving in) and at T2 (one year later).

Health	Adults Test Group			Adults Control Group		
	Before Moving	T1	T2	Before Moving	T1	T2
Excellent	18.9	25.2	24.5	16.3	20.8	18.7
Very good	41.4	50.5	50.9	46.9	52.5	51.6
Good and less good	39.6	24.3	24.5	35.6	26.7	29.7
Poor	-	-	-	1.2	-	-

Chi^2^ test: test vs. control group: Before moving: *p* = 0.472; T1: *p* = 0.621; T2: *p* = 0.735.

**Table 2:** Parental rating of their children’s health (percentages) at T1 (three months after moving in) and at T2 (one year later).

Health	Children Test Group		Children Control Group	
	T1	T2	T1	T2
Excellent	60.5	41.5	28.2	50.0
Very good	21.1	43.9	53.9	41.2
Good and less good	18.4	14.6	17.9	8.8

Chi^2^ test: test vs. control group: T1: *p* = 0.006; T2: *p* = 0.086.

**Table 3:** Prevalence of symptoms or health impairments (“always” and “often”) in both groups at both measuring time points (T1: three months after moving in; T2: one year later).

Symptom or Complaint, %	Test Group T1	Control GroupT1	Test GroupT2	Control GroupT2
Tiredness	65.5	67.9	72.8	61.6
Exhaustion	42.5	50.3	51.6	46.5
Headache	29.5	40.8	29.4	34.9
Mood change	32.9	34.0	34.9	35.3
Anxiety	26.0	30.6	26.8	31.0
Limited performance	21.2	22.4	21.6	30.2
Nervousness	16.4	19.7	24.0	24.2
Impaired concentration	20.5	21.1	17.6	23.3
Nausea	10.3	10.2	6.5	9.3
Dizziness	8.9	11.6	7.8	7.8
Complaints (average number)	2.7	3.1	2.9	3.0

**Table 4:** Negative perception of indoor air quality in both groups at T1 (three months after moving in) and T2 (one year later).

Air Quality: Negative Attributes, % *	Test Group T1	Control Group T1	Test Group T2	Control Group T2
Stale	14.0	37.8	22.7	38.5
Stuffy	12.1	26.5	10.9	22.0
Stagnant	14.0	42.9	10.9	45.1
Bad smelling	5.6	11.2	1.8	3.3
Smoky	0.9	1.0	2.7	2.8

* Answer categories 2–5 (“a little” to “predominantly”) are combined (percentages).

**Table 5:** Positive perception of indoor air quality in both groups at T1 (three months after moving in) and T2 (one year later).

Air Quality: Positive Attributes, % *	Test Group T1	Control Group T1	Test Group T2	Control Group T2
Pleasant	49.5	28.6	45.5	25.3
Clean	44.9	32.7	40.9	27.5
Fresh	39.3	14.3	32.7	9.9
Fragrant	0.0	1.0	1.8	1.1

* Percentages are related to answer category 5 (“predominantly”).

**Table 6:** Perception of indoor climate, smell and noise. Percentage of participants who answered with “just right” regarding room temperature, humidity, air movement or “not annoying at all” regarding smell and noise. T1 (three months after moving in) and T2 (one year later).

Just Right/Not Annoying At All, %	Test Group T1	Control Group T1	Test Group T2	Control Group T2
Room temperature	77.6	61.2	76.4	69.2
Humidity	63.9	65.3	53.6	69.2
Air movement	81.3	68.4	80.0	64.8
Smell	51.4	49.0	49.1	56.0
Noise	56.1	55.1	48.2	56.0

**Table 7:** Satisfaction with the housing situation in both groups T1 (three months after moving in) and T2 (one year later).

Satisfaction, %	Test Group T1	Control Group T1	Test Group T2	Control Group T2
Very content	86.9	76.5	81.8	75.8
Content	10.3	21.5	15.5	23.1
Neither more nor less content/dissatisfied	2.8	2.0	2.7	1.1

## Abbreviations

The following abbreviations are used in this manuscript:

T1–T2	first interview and first measuring point three months after moving in (T1) resp. follow-up interview and second measuring point one year later (T2)
